# Are patients with a nasally placed feeding tube at risk of potential drug-drug interactions? A multicentre cross-sectional study

**DOI:** 10.1371/journal.pone.0220248

**Published:** 2019-07-31

**Authors:** Fernanda Raphael Escobar Gimenes, Melissa Baysari, Scott Walter, Leticia Alves Moreira, Rhanna Emanuela Fontenele Lima de Carvalho, Adriana Inocenti Miasso, Fabiana Faleiros, Johanna Westbrook

**Affiliations:** 1 Department of General and Specialized Nursing, University of São Paulo at Ribeirão Preto College of Nursing, Ribeirão Preto, São Paulo, Brazil; 2 Centre for Health Systems and Safety Research, Australian Institute for Health Innovation, Macquarie University, Sydney, NSW, Australia; 3 Ceará State University, Fortaleza, Ceará, Brazil; 4 Department of Psychiatric Nursing and Human Sciences, University of São Paulo at Ribeirão Preto College of Nursing, Ribeirão Preto, São Paulo, Brazil; University of Notre Dame Australia, AUSTRALIA

## Abstract

**Aims:**

The primary aims were to determine the rate of potential drug-drug interactions (pDDIs) in patients with nasally placed feeding tubes (NPFT) and the factors significantly associated with pDDIs. The secondary aim was to assess the change in pDDIs for patients between admission and discharge.

**Material and methods:**

This multicentre study applied a cross-sectional design and was conducted in six Brazilian hospitals, from October 2016 to July 2018. Data from patients with NPFT were collected through electronic forms. All regular medications prescribed were recorded. Medications were classified according to the World Health Organization (WHO) Anatomical Therapeutic Chemical code. Drug-drug interaction screening software was used to screen patients’ medications for pDDIs. Negative binomial regression was used to account for the over dispersed nature of the pDDI count. Since the number of pDDIs was closely related to the number of prescribed medications, we modelled the rate of pDDIs with the count of pDDIs as the numerator and the number of prescribed medications as the denominator; six variables were considered for inclusion: time (admission or discharge), patient age, patient gender, age-adjusted Charlson Comorbidity Index (CCI) score, type of prescription (electronic or handwritten) and patient care complexity. To account for correlation within the two time points (admission and discharge) for each patient a generalised estimating equations approach was used to adjust the standard error estimates. To test the change in pDDI rate between admission and discharge a full model of six variables was fitted to generate an adjusted estimate.

**Results:**

In this study, 327 patients were included. At least one pDDI was found in more than 91% of patients on admission and discharge and most of these pDDIs were classified as major severity. Three factors were significantly associated with the rate of pDDIs per medication: patient age, patient care complexity and prescription type (handwritten vs electronic). There was no evidence of a difference in pDDI rate between admission and discharge.

**Conclusion:**

Patients with a NPFT are at high risk of pDDIs. Drug interaction screening tools and computerized clinical decision support systems could be effective risk mitigation strategies for this patient group.

## Introduction

Many patients receive enteral nutrition, liquid and medications through short-term enteral access devices. The number of patients with a nasally placed feeding tube (NPFT) in hospitals and at home is increasing worldwide due to the increasing number of older adults with Alzheimer disease or other dementias, patients with poor swallowing reflexes and nutritional status, and the shift in care provision from acute to community settings [1–3]. However, the majority of tube-fed patients are on medications that require special precautions, thus posing pharmaceutical challenges in the delivery of medications usually delivered by the oral route [4]. For instance, patients using more than five drugs have greater chance of having their NPFT exchanged due to clogging [5]. In addition, most of these patients suffer from various comorbidities and the complex polypharmacotherapy used to treat their chronic diseases makes patients with NPFT vulnerable to drug-drug interactions (DDIs) (typically defined as a change in the effect of a drug when it is taken together with another drug and may involve an increase in the action of either drug, a decrease in drug efficacy, a delay in drug absorption rate, or an unexpected harmful side effect) [6].

DDIs in susceptible hospitalized patients are one of the major causes of preventable adverse drug events caused by drugs [7]. DDIs account for up to 5% of hospital admissions per year, can lead to an increase in the length of hospital stay, and result in higher costs associated with healthcare [8].

The prevalence of potential drug-drug interactions (pDDIs) in general inpatients has been reported to be between 16.3% and 71.1%, with a mean range of 0.3 to 4.5 per patient [9]. Drug regimen changes during hospital admission or discharge have also been associated with an increased number of pDDIs [10,11], exposing patients to significant risks.

The consequences of a DDI are an important safety concern. Identifying patients with pDDIs might optimise the allocation of targeted care and improve patient outcomes.

Several single-centre studies have identified the prevalence of pDDIs in general inpatients, however, there are no studies reporting on pDDIs rates among patients with NPFT across multiple hospital sites and at a national level. Furthermore, previous studies have not evaluated the use of risk-adjustment measures that would allow adjustment for confounders for the occurrence of pDDIs on admission and discharge.

The primary aims of this study were to determine the rate of pDDIs in patients with NPFT and the factors significantly associated with pDDIs, with potential factors including regimen change (admission or discharge), patient age, patient gender, age-adjusted Charlson Comorbidity Index (CCI) score, type of prescription (electronic or handwritten) and patient care complexity. The secondary aim was to assess the change in pDDI rate for patients between admission and discharge, adjusted for other potentially confounding factors.

## Materials and methods

### Study design

This paper is part of a broader research project on feeding tube-related incidents. This was a multicentre study with a cross-sectional design.

### Setting

Six centres across Brazil participated in this study; the centres included a mix of community and university hospitals, hospitals with and without residency programs, and public and private hospitals; they also varied in size. The hospitals were as follows: Acre Hospital of Clinics—HCA (Acre), General Hospital of Fortaleza—HGF (Ceará), Hospital of Clinics of the Medical School of Ribeirão Preto of the University of São Paulo—HCFMRP-USP (São Paulo), Américo Brasiliense State Hospital—HEAB (São Paulo), São Vicente de Paulo Hospital—HSVP (Minas Gerais), and Santa Cruz Hospital—HSCRGS (Rio Grande do Sul). The medical wards of these hospitals were chosen for this study because many adult patients in these wards have chronic conditions and require enteral nutrition and medications through NPFT.

Three of the hospitals use an electronic system for prescribing, however the systems do not include any decision support for DDIs (e.g. DDI alerts). Monitoring of patients’ profiles for pDDIs is not part of routine clinical practice in the study hospitals.

### Participants

The inclusion criteria were patients older than 18 years; who were admitted to an internal medical ward with a nasally placed gastric tube or small-bowel feeding tube (or patients who required the insertion of these tubes during hospitalization); and patients that were hospitalized for at least 24 hours. Patients meeting the above inclusion criteria who were re-admitted during the study period were only counted for their first admission.

Sample size was determined by stratified random sampling with proportional allocation by strata, where each stratum was formed by the units of each hospital. Adopting the parameters of relative error of 20%, level of significance of 5% and the total population of 4,573 patients with a short-term NPFT in a period of six months, a total sample size of 281 patients was calculated.

### Instruments

Data collection tools were composed of electronic forms which were developed by the research team, and assessed for face and content validity by a panel of experts. The forms were developed in the Portuguese language using an online platform (Survey Monkey). The experts were selected through an analysis of existing curricula in the database of the Brazilian National Council for Scientific and Technological Development (CNPq) and were invited to provide their expertise on the design of the forms. Links to the electronic forms were made available to experts to obtain consensus and to determine the final content of the forms. The modified forms were pilot tested prior to being finalized. This involved the forms being applied to five hospitalized patients from the first day of use of NPFT until patient discharge.

### Database used for pDDI detection and data collection

Data were collected from October 2016 to July 2018. All regular medications prescribed were recorded within the first 24 hours after admission and within 24 hours before discharge, regardless of whether medications were administered or not. When a patient was prescribed a drug in different dosing regimens (e.g. rapid- and moderate-acting insulin), the agent was counted only once [12]. We excluded medications given topically (such as enemas, eye drops, creams, gels, moisturizers, nicotine, nystatin) and contrast prescriptions (i.e. contrast for cerebral angiography).

Potential interactions between drugs and enteral nutrition or foods were not assessed, because they were beyond the scope of this study. Drugs which were prescribed more than once for the same patient were counted only once. A drug-drug interaction screening software [13] was used to screen patients’ profiles for pDDIs because it is a highly reliable software for detecting DDIs, which possesses sufficient sensitivity (≥ 83%) and specificity (≥ 90%) [14]. In addition, this software is available in the journal portal of the Coordination of Improvement of Higher-Level Personnel—CAPES, Brazil, and thus it is easily accessed by hospital staff.

All drugs in a patient’s medication profile were entered one by one into the DDI screening software. The software displays all interacting combination(s) present in the medication profile. It also provides information about the mechanism and potential adverse outcomes of an interaction [15]. All identified pDDIs were categorized on the basis of their levels of onset (immediate, rapid, delayed, and unknown), severity (contraindicated, major, moderate, minor and unknown), and documentation (excellent, good, fair, and unknown), as described by the software [16].

The primary reason for hospital admission was coded according to the World Health Organization (WHO) International Classification of Diseases, 10th revision (ICD-10). Severity of comorbid diseases was evaluated using the CCI [17]. It measures the severity of the patient, regardless of the main diagnosis, and it is able to predict the one-year mortality of patients. The final score is the result of the sum of the weights assigned to the comorbidities recorded as secondary diagnoses; the higher the score, the greater the risk of the patient dying. In our study, the CCI was adjusted according to the patient’s age, so that, from the age of 50, a point was added to the final score for every decade of life [17]. Based on the final age-adjusted CCI score, patients were divided into three groups: mild risk of death (with CCI scores of 1–2); moderate risk of death (with CCI scores of 3–4); and severe risk of death (with CCI scores ≥ 5) [18].

Patient care complexity was assessed by an experienced nurse, using the Patient Classification System (PCS) [19]. The PCS is recommended by the Federal Nursing Council, Brazil [20], and it was developed to classify patients according to the degree of dependence on nursing care. This instrument has nine critical indicators: mental state, oxygenation, vital signs, mobility, ambulation, feeding, body care, elimination and therapeutics. Therefore, the scores are distributed in five categories that correspond to the complexity of nursing assistance: minimum care (scores of 9–14), intermediate care (scores of 15–20), high-dependency care (scores of 21–26), semi-intensive care (scores of 27–31), and intensive care (scores > 31) [19].

### Data analysis

Patient-related data were downloaded from the Survey Monkey platform into a computer file by the principal investigator. Data were analysed using the Statistical Package for the Social Program Sciences (SPSS) version 22.0 (SPSS Inc., Chicago, IL, USA) and version 9.4 of the SAS system for Windows (SAS Institute Inc., Cary, NC, USA). For data analyses, medications were also classified according to the WHO Anatomical Therapeutic Chemical (ATC) code.

Since the number of pDDIs was closely related to the number of prescribed medications, we modelled the rate of pDDIs with the count of pDDIs as the numerator and the number of prescribed medications as the denominator. This quantifies the pDDI risk independent of the number of prescribed medications and was done through negative binomial regression to account for the over dispersed nature of the pDDI count. Six variables were considered for inclusion: time (admission or discharge), patient age, patient gender, age-adjusted CCI score, type of prescription (electronic or handwritten) and patient care complexity. All variables were initially included and were subsequently removed one by one using a backwards change-in-estimates approach [21]. Any variable removal causing less than a 20% change in all remaining estimates was deleted. To account for correlation within the two time points for each patient a generalised estimating equations approach was used to adjust the standard error estimates. Of the 654 data points (two per patient), 9 were omitted from the model due to missing covariate values. A plot of deviance residuals against predicted values indicated approximate symmetry around zero. The ratio of deviance to degrees of freedom was not significantly different from one (p = 0.96).

To test the change in pDDI rate between admission and discharge a full model of all six variables was fitted to generate an adjusted estimate. Again, the rate was modelled with negative binomial regression, but this time a random intercepts approach was used to generate a subject specific estimate of the change over time.

### Ethics

The study was approved by the Research Ethics Committee of the University of São Paulo at Ribeirão Preto College of Nursing, according to Resolution No. 466/2012, of the National Council of Ethics in Research of the Brazilian Ministry of Health, which addresses research ethics with humans (CAAE: 56166016.1001.5393) and written informed consent was obtained from each patient, or their guardian, prior to enrolment in the study.

## Results

327 patients with NPFT were included in the analyses. From those, 183 (56.0%) were admitted withouth a NPFT and 144 (44.0%) were admitted with a NPFT. The patient sample consisted mostly of males (176, 53.8%), was elderly (aged 60 years or more), with a mean length of hospital stay of 18 days. The main reasons for hospitalization were “IX Diseases of the circulatory system” (76, 23.2%), “II Neoplasm” (53, 16.2%), and “X Diseases of the respiratory system” (40, 12.2%). The most common comorbidity was peripheral vascular disease (81, 24.8%), followed by cerebrovascular disease (57, 17.4%), diabetes without complication (40, 12.2%) and metastatic solid tumour (35, 10.7%). Most patients had at least one comorbidity (122, 67.9%), were at severe risk of death (141, 43.1%), with a mean age-adjusted CCI score of 4.25 (± 2.77), and were highly dependent on nurse care (115, 35.4%) (Table 1) (S1 Table).

**Table 1:** Patient characteristics (N = 327).

Variables	N patients	%
**Patient Age**		
<55	92	28.1
55 − <65	58	17.7
65 − <75	89	27.2
≥75	88	26.9
**Gender**		
Male	176	53.8
Female	151	46.2
**The three-primary reason for hospital admission—ICD-10**		
IX Diseases of the circulatory system	76	23.2
II Neoplasms	53	16.2
X Diseases of the respiratory system	40	12.2
**The four most frequent comorbidities**^a^		
Peripheral vascular disease	81	24.8
Cerebrovascular disease	57	17.4
Diabetes without complication	40	12.2
Metastatic solid tumor	35	10.7
**Age-Adjusted Charlson Comorbidity Index (CCI)**		
Severe risk of death (with CCI scores ≥ 5)	141	43.1
Moderate risk of death (with CCI scores of 3–4)	92	28.1
Mild risk of death (with CCI scores of 1–2)	64	19.6
No risk of death (with CCI score equal to 0)	29	8.9
**Patient Care Complexity**		
High-dependency (scores of 21–26)	115	35.2
Intermediate (scores of 15–20)	86	26.3
Minimum (scores of 9–14)	53	16.2
Semi-intensive (scores of 27–31)	52	15.9
Intensive (scores > 31)	14	4.3

SD, standard deviation; ICD-10, International Classification of Diseases, 10th revision; CCI, Charlson Comorbidity Index.

^a^Patients could have more than one comorbidity.

The mean number of medications prescribed, per patient, using the electronic system was higher on admission (mean 9.86 medications, SD 3.56) and discharge (mean 10.10 medications, SD 3.73), when compared to handwritten prescriptions (mean 7.36 medications, SD 3.50 and mean 6.40 medications, SD 4.08, respectively).

Patients had 2,057 pDDIs on admission (mean 6 pDDIs, SD 5.51) and 2,232 pDDIs on discharge (mean 7 pDDIs, SD 6.57). At least one pDDI was found in 307 (93.9%) patients on admission and in 299 (91.4%) patients on discharge. We identified 581 and 632 different drug pairs on admission and discharge respectively (Table 2) (S2 Table).

**Table 2:** Characteristics of potential drug-drug interactions (pDDIs) on admission (N = 2,057) and discharge (N = 2,232).

Characteristics	Admission		Discharge	
	n	%	n	%
Patients with ≥ 1 pDDIs	307	93.9	299	91.4
pDDI per patient—median ± SD (range)	6 ± 5.51 (0–26)	-	7 ± 6.57 (0–34)	-
**Time of Onset of pDDIs**				
Not Specified	1.604	78	1,733	77.6
Delayed	341	16.6	395	17.7
Rapid	110	5.3	104	4.7
Unknown	1	0	0	0
**Severity of pDDIs**				
Major	1,309	63.7	1,427	63.9
Moderate	574	27.9	611	27.4
Contraindicated	108	5.2	131	5.9
Minor	64	3.1	63	2.8
Unknown	1	0	0	0
**Documentation of pDDIs**				
Fair	1,251	60.8	1,406	63
Good	506	24.6	520	23.3
Excellent	298	14.5	306	13.7
Unknown	1	0	0	0

SD, standard deviation

In 71 patients (21.7%), the number of pDDIs remained the same on admission and discharge; 122 (37.3%) patients experienced fewer pDDIs on discharge compared to admission, while the remaining patients (134, 41%) had more pDDIs on discharge than admission, although 106 (79.1%) of these patients had more medications prescribed on discharge.

The onset of the pDDIs was classified as delayed in 341 (16.6%) on admission and in 395 (17.7%) on discharge. In relation to the severity of pDDIs, more than 63% were classified as major, both on admission (1,309, 63.7%) and discharge (1,427, 63.9%). With respect to documentation, the majority of pDDIs identified during both admission and discharge were considered fair (Table 3) (S2 Table).

**Table 3:** The ten most common pDDIs in patients with NPFT on admission (N = 2,056) and discharge (N = 2,232).

Drug-Drug Combination	Severity	Documentation	Frequency	
			n	%
**Admission (N = 2,056)**				
Dipyrone -- Heparin Sodium	Major	Fair	83	4
Dipyrone -- Enoxaparin Sodium	Major	Good	82	4
Aspirin -- Dipyrone	Major	Excellent	66	3.2
Bromopride -- Tramadol Hydrochloride	Major	Fair	44	2.1
Insulin Human Regular -- Metoclopramide	Major	Fair	41	2
Dipyrone -- Furosemide	Major	Good	36	1.7
Aspirin -- Heparin Sodium	Major	Fair	30	1.5
Dipyrone -- Losartan Potassium	Moderate	Excellent	29	1.4
Aspirin -- Insulin Human Regular	Moderate	Fair	25	1.2
Morphine Sulfate -- Ondansetron	Major	Fair	24	1.2
**Discharge (N = 2,232)**				
Dipyrone -- Heparin Sodium	Major	Fair	78	3.5
Dipyrone -- Enoxaparin Sodium	Major	Good	62	2.8
Aspirin -- Dipyrone	Major	Excellent	48	2.1
Dipyrone -- Furosemide	Major	Good	40	1.8
Bromopride -- Morphine Sulfate	Major	Fair	38	1.7
Insulin Human Regular -- Metoclopramide	Major	Fair	35	1.6
Dipyrone -- Sertraline Hydrochloride	Major	Excellent	34	1.5
Bromopride -- Tramadol Hydrochloride	Major	Fair	33	1.5
Morphine Sulfate -- Ondansetron	Major	Fair	26	1.2
Aspirin -- Heparin Sodium	Major	Fair	25	1.1

The rate of pDDIs per medication was lower for older age groups compared to those aged less than 55, with patients aged 65 or older being significantly lower (rate ratios [RR] 0.78 or 0.80) (Table 4) (S3 Table). The rate was higher for patients with a non-zero (≥1) age-adjusted CCI score, but this was not significant, although the variable overall met the inclusion criteria for the model. Where health facilities used an electronic prescribing system, the rate of pDDIs was 62% higher on average than handwritten prescribing systems (RR 1.62, 95% CI 1.38, 1.90). Compared to patients requiring minimal care, the pDDI rate was significantly higher for all other levels of patient complexity, with the highest for high-dependency care (RR 1.44, 95% CI 1.18, 1.75).

**Table 4:** Estimated ratios of pDDI rate per prescribed medication in patients with NPFT, from a negative binomial regression model.

Variable	N patients^a^	Rate ratio (95% CI)	p-value
**Patient age**			
<55 (reference)	92	1	
55 − <65	58	0.91 (0.76, 1.10)	0.35
65 − <75	89	0.78 (0.65, 0.94)	0.01
≥75	88	0.80 (0.66, 0.96)	0.02
**Age-adjusted CCI**			
0 (reference)	29	1	
1–2	66	1.15 (0.91, 1.46)	0.23
3–4	90	1.18 (0.90, 1.55)	0.22
≥5	141	1.18 (0.91, 1.52)	0.22
**Prescription type**			
Handwritten (reference)	72	1	
Electronic	255	1.62 (1.38, 1.90)	<0.001
**Patient care complexity**			
Minimal care (reference)	53	1	
Intermediate Care	86	1.28 (1.04, 1.57)	0.02
Semi-intensive care	52	1.40 (1.12, 1.74)	0.003
Intensive Care	14	1.40 (1.09, 1.80)	0.01
High-dependency care	115	1.44 (1.18, 1.75)	<0.001

CI, confidence interval; CCI, Charlson Comorbidity Index.

^a^For age-adjusted CCI score and patient care complexity, counts do not sum to the total of 327 patients due to missing values for 1 and 2 patients, respectively.

The crude rate of pDDIs per prescribed medication was 0.68 on admission and 0.73 on discharge. The number of prescribed medications was very similar on admission and discharge, with means of 9.31 and 9.29, respectively. The mean difference between numbers of medications on admission and discharge was -0.02. The adjusted estimate of the difference in rate of pDDIs showed that the rate was 5% higher on discharge compared to admission (rate ratio 1.05, 95% CI 0.98, 1.12), but this was not a significant difference (p = 0.14).

## Discussion

The occurrence of pDDIs in patients with NPFT found in this study was much higher (more than 90% of patients) than those in other studies for patients from internal medical wards (from 34.4% to 78.2%), both on admission and discharge [11,15,22–26]. In our study, over 60% of all pDDIs were classified as major severity, which is higher than in other studies of both inpatients [22,23,26] and outpatients [27]. All patients require close surveillance for pDDIs, especially those patients with NPFT. In this study, patients with NPFT were taking a large number of medications on admission and discharge, with a mean of 9.31 and 9.29, respectively. These results are higher than those found in previous studies conducted in general medicine wards (4–7 medications) [22–26,28], thus healthcare professionals should be aware of this risk and use effective risk mitigation strategies for this patient group in order to improve patient outcomes.

DDIs, when they occur, can lead to increased hospital stay and to increased overall health care costs, thus they should be checked in patients with NPFT, as this can be effective in reducing adverse drug events and in improving patient outcomes.

We found that the risk of pDDI per medication approximately decreased with age. Modelling the pDDI count, as done in some other studies, showed a similar result [22]. The variable "age" is used in several risk stratification tools, but results from previous studies showed that its impact on increased risk for pDDIs is still unclear. For instance, studies conducted in an emergency department in the Caribbean [29] and internal medicine wards in hospitals in Pakistan [15] and South India [22] showed that older patients (age of 60 years or more) were more likely to have pDDIs. While, a study conducted in the internal medicine ward of Tikur Anbessa specialized hospital found no association between patient age and exposure to pDDIs [26]. Thus, a plausible explanation for our result could be the influence of other risk factors that may increase younger patients’ exposure to pDDI, including drug class prescribed, multiple prescribers, the primary reason for hospital admission, and severity of comorbidities [30–32]. However, true causes of this phenomenon remain to be elucidated by future, targeted research.

In our study, we found no strong evidence that the age-adjusted CCI score categories affect the rate of pDDI, and this effect persisted when CCI was included as binary (0 vs. 1 or more) or continuous. However, it met the inclusion criteria of the model, indicating that it is a confounder of other variables. Previous studies have showed that the CCI score is a risk factor of pDDIs [32, 33], but applying their analysis methods to our data the CCI effect remained non-significant.

Compared to patients requiring minimal care, the pDDI rate was significantly higher for all other levels of patient care complexity, with the highest rate observed for high-dependency or intensive care patients. These patient groups had more prescribed medications and greater pDDI risk per medication, making them considerably more vulnerable to the occurrence of any pDDI. We found no studies correlating the Brazilian system used to classify patients according to the degree of dependence on nursing with risk of pDDIs; however, previous studies have shown that critical care patients are at increased risk of pDDIs because they present with severe and life-threatening illnesses. Most of them suffer from various comorbidities, and they usually receive complex pharmacotherapy which increases the risk of DDIs [32,34–36].

Where health facilities used an electronic prescribing system, the rate of pDDIs per prescribed medication was 62% higher on average than handwritten prescribing systems. In addition, the electronic system had both more medications prescribed per patient and more pDDIs per medication, representing an even higher risk of patient experiencing any pDDI. The number of medications prescribed tends to be lower in handwritten systems (5.23–8.2 medications) [37–39] compared to facilities using electronic prescribing systems (8.9–10 medications) [12,40], as was the case in our study. Thus, the increased number of medications in electronic system combined with the increased risk of pDDI per medication indicates an area of concern for such systems as contributors to pDDI risk. Experts call attention to the importance of the implementation of electronic prescribing systems with computerized alerts for this purpose [41–43]. However, these alerts can be disruptive and can become sources of annoyance and frustration when used instinctively, resulting in automation bias and leading to decision errors [9]. Opportunities to improve DDI alerting include using differential displays based on severity, establishing improved lists of clinically significant DDIs, and thoroughly reviewing organizational implementation decisions regarding DDIs. Alerts properly developed and implemented have the potential to prevent adverse drug events and improve patient safety, thus, institutions should carefully review their DDIs alerting approaches and a national and international alerting strategy for DDIs should be considered [7,44,45].

The adjusted rate of pDDIs per prescribed medication was non-significantly higher at discharge compared to admission. Few studies have compared the occurrence of pDDIs at these two time points (admission and discharge), but those that have, showed an increase in the proportion of patients with any pDDI on discharge [11,46]. However, these effects were related to a corresponding increase in the number of prescribed medications on discharge, and it is not clear that there was an increase in the risk of pDDI per medication in both time points.

It is expected that patients’ drug regimens change during hospitalisation particularly when they are seriously ill or are deteriorating [38]. However, patients’ exposure to pDDIs on discharge raises a safety concern. A previous study of DDIs in a cohort of hospitalized elderly patients in Milan, Italy, found a significant association with mortality at 3 months after discharge in patients with at least two potentially severe DDIs during hospitalization [47]. A retrospective study of 1,487 adult patients admitted to a general hospital in the North-eastern region, Brazil, suggested an association between prior drug interactions and risk of hospital readmission [48].

In medical practice, it is quite common to intentionally use drug combinations that may interact because the risk-benefit may be positive to treat patients with complex healthcare needs. Therefore, the decisions taken by doctors to minimize pDDIs’ impact on their patients should be determined on an individual basis, and this requires careful evaluation of the risk-benefit ratio between treatment discontinuation vs. continuation but with close monitoring [49].

Potential DDIs in patients with NPFT may be due to the reduced formulary of medications available that are suitable to be used with NPFT, as is the case in many Brazilian hospitals. Thus, it can be difficult for prescribers to find a non-interacting drug for administration to a patient with NPFT. Prescribers may consider other form selection and routes, such as endovenous, transdermal, rectal or topical [50], although not all drugs are available via these routes.

Despite there are pDDIs, this risk may be clinically necessary in order to treat the patient’s clinical conditions, for example, there may be a pDDI with a medication and an antibiotic for pneumonia. However, the risk of not treating pneumonia outweighs the risk of the pDDI. Physicians, pharmacists, and nurses should be aware of possible factors that may increase the risk for DDIs in hospitalized patients. The results of this study show that patients with NPFT need an individualized and more rigorous evaluation of their drug therapy, especially patients that require intensive care. Thus, appropriate patient monitoring and clinical management procedures are essential for minimizing and preventing potential adverse outcomes [34], including periodic evaluation of the patients’ drug regimens, dose adjustments and dosing changes, control of the drugs’ serum levels, and monitoring of patients’ clinical conditions [51,52].

## Limitations and strengths

All drugs prescribed on admission and discharge were included in the analysis, whether they were administered or not, which may have increased the rate of pDDIs. Although dose may impact interactions, duplicated agents were excluded from our analyses. However, our findings provide a baseline rate of pDDIs and useful information on factors related to pDDIs in hospitalized medical ward patients with NPFT. To our knowledge, this is the first large scale study in Brazil and in Latin America documenting the prevalence of pDDIs in internal medicine wards. It is also the first study to examine DDIs in patients with NPFT and to analyse pDDIs as a rate per prescribed medication.

## Conclusion

More than 90% of patients with NPFT are exposed to at least one pDDI during their hospital stay and most of them are severe. In addition, the average number of pDDIs per patient was high. Factors associated with risk of pDDI per prescribed medication comprised age, high care complexity and the use of electronic prescribing systems in care facilities. There was no evidence of a difference in rate of pDDIs per prescribed medication between admission and discharge.

Drug interaction screening tools and computerized clinical decision support systems can be effective risk mitigation strategies for potentially relevant DDIs in patients with NPFT. However, it is equally important to empower front-line healthcare professionals to monitor for pDDIs, especially pharmacists and nurses, to raise awareness and prevent harm to patients who are at risk of pDDIs. Future work is needed to determine the real harm pDDIs inflict on patients.

## Supporting information

S1 Table Dataset. Patients’ data on admission and discharge.
(XLSX)

S2 Table Dataset. Data on medications prescribed to patients with NPFT on admission and discharge.
(XLSX)

S3 Table Statistical analysis. Supporting information on data analyses.
(XLSX)
